# Temporal modulation of tactile perception during balance control

**DOI:** 10.1038/s41598-025-99006-8

**Published:** 2025-05-19

**Authors:** Fabian Dominik Wachsmann, Katja Fiehler, Dimitris Voudouris

**Affiliations:** Experimental Psychology, Justus Liebig University Giessen, Otto-Behaghel-Str. 10 F, 35394 Giessen, Germany

**Keywords:** Perception & action, Postural adjustments, Tactile modulation, Virtual reality, Human behaviour, Cognitive neuroscience, Motor control, Sensorimotor processing, Sensory processing

## Abstract

Somatosensory feedback, like touch, is essential for body control and movement. Yet tactile sensations from a body part that is about to move or is moving are often suppressed. Most studies on tactile suppression focus on upper-limb movements, where suppression is typically reduced when tactile signals become important to the task. However, how tactile sensitivity changes at other body parts involved in more complex, whole-body actions is widely unexplored. This study examines the temporal tuning of tactile processing on the lower limb during balance control while varying feedback processing demands. Participants stood in a virtual room, with the front wall moving toward them at a moment of high or low temporal uncertainty challenging their posture. Tactile sensitivity was probed using vibrotactile stimuli at the lower leg at different time points relative to perturbation onset. We found that tactile sensitivity while standing improved around the time of perturbation, irrespective of the temporal uncertainty about perturbation onset. Such dynamic modulation indicates a continuous process of sensory feedback regulation to accomplish the task at hand. This expands previous knowledge about sensory integration on the lower limbs during postural control.

## Introduction

Tactile sensitivity on a moving limb compared to the same limb in a static state is reduced. This is typically reflected in poorer sensitivity to externally-generated probe tactile stimuli that are delivered to a limb while being engaged in movement planning or execution. This tactile suppression is shown in simple finger extensions, and more complex upper-limb actions such as reaching, grasping and even juggling^1–5^, with reduced tactile sensitivity observed at both perceptual^1,4,5^and neural levels^6–8^. Although tactile suppression can be partly explained by peripheral mechanisms, such as additional reafferent signals masking the probe tactile stimuli^2,9^, see also^10^, the prevailing idea is that sensorimotor predictions down-weight the incoming sensory feedback arising from the movement^11,12^.

Tactile suppression of vibrotactile probes on a moving limb can be modulated by various factors. For instance, when the sensations arising on a moving limb are highly predictable, tactile suppression on that limb increases^2,13^. On the other hand, tactile suppression is reduced on a moving limb when sensory feedback from that limb is needed to accomplish the task. For instance, tactile suppression on the index finger of a grasping hand is reduced when grasping objects of unknown properties or of low friction^14,15^, reflecting that the increased need to sample tactile information for accomplishing the motor task allows the sensorimotor system to tune tactile suppression of afferent signals. The tuning of tactile sensitivity on a moving limb is also reflected in dynamic temporal changes during the execution of arm movements^16–18^. Indeed, tactile suppression is reduced at moments of the reaching phase when sensory guidance of the arm’s movement gains importance^18^. All in all, these results reflect that tactile sensitivity on a body part largely depends on the dynamic interplay between central predictive and somatosensory feedback signals.

Despite the large body of research on the modulation of tactile processing, mechanisms underlying up- and down-regulation of tactile signals during whole body movements are still unclear. For instance, some studies found that somatosensory-evoked potentials (SSEPs) related to tactile stimulation on the foot sole are decreased prior to a foot flexion^[e.g.19^. However, other studies revealed an opposite pattern with increased SSEPs prior to step initiation^20^. This apparent discrepancy in physiological signal processing may arise from the different needs to use tactile feedback for the actions in question, as has been proposed for upper limb movements. Specifically, prior to initiating a step^20^, tactile sensitivity on the foot sole may improve as tactile feedback from that body part provides crucial information for balance control. In contrast, tactile feedback from the foot sole prior to an arbitrary foot flexion^19^ may be less relevant for movement execution and thus it may be down-regulated. The modulation of tactile sensitivity during postural control is not surprising as it is observed in various instances and across various sensory modalities. For instance, H- and T-reflexes are inhibited when postural demands increase^21,22^, presumably as a protective mechanism that facilitates balance. In addition, vestibular signals are suppressed when artificial delays are introduced between the generation of ankle torques when standing and the consequent whole-body responses^23^. However, when participants are exposed for long enough to learn the coupling between the generation of the torques and the delayed sensorimotor consequences, vestibular signal integration recovers^23^. Another example for vestibular reweighting can be observed during postural transitions from standing to locomotion with a reduction of vestibular signal processing during the transition phase^24^. Modulation of other sensory signals, such as proprioceptive signals, can also be observed when anticipating the avoidance of a virtual ball. Visual signals are up-weighted while proprioceptive signals from the lower leg are down-weighted, though this modulation can be reversed with more exposure to the task^25^. Considering that processing of sensory signals related to upright stance is modulated by the task dynamics, we are here interested in examining how tactile processing is tuned when confronted with various postural demands. Specifically, we test the hypothesis that tactile sensitivity is modulated by the need to process tactile feedback from the lower leg that is involved in the retention of upright posture. We applied a common psychophysical approach allowing us to compare our results to previous work on tactile suppression during upper limb movements^13,26^.

Tactile information is important for maintaining upright posture. For instance, tactile signals from the foot sole, including plantar cutaneous afferents, convey spatiotemporal information about skin stretch and pressure variations^27,28^, and these signals can be used to adjust body posture accordingly. Tactile signals from the foot sole are also integrated prior to the initiation of an upcoming step^29^, whereas disrupting the quality of tactile information from the foot sole can result in poorer upright stance^30,31^. However, tactile information from other body parts can also play an important role for maintaining upright stance. A common instance refers to tactile signals that arise when lightly touching a surface with the finger or hand, which can facilitate postural sway, even when touch force levels do not physically support the body^32,33^. This light touch could provide somatosensory (i.e., tactile and proprioceptive) signals about arm position, which could inform the postural system about necessary adjustments, and even down-weight the contribution of other sensory inputs, such as electrical vestibular stimulation, which could disturb balance^34^. However, light-touch is not the only touch-related input that can influence balance control, as passively obtained tactile information can also facilitate posture. Specifically, rubbing a piece of fabric at the skin over the shoulder area can lead to reduced postural sway in young, older and pathological populations^35^. Similar results have been found when rubbing tactile stimuli (e.g., Velcro materials) at various positions of the lower leg, such as between the knee and the ankle. In sum, several studies have shown that tactile signals from various body parts, including the lower legs, can provide crucial information to support body balance^36^ and to obtain a better estimate of the current body configuration to facilitate postural adjustments. Based on these, if tactile processing is modulated at moments when somatosensory control of posture gains importance, we expect improved tactile sensitivity on the lower leg at moments when tactile input can be useful for postural control, such as before the onset of a postural adjustment in response to a perturbation.

To examine how tactile sensitivity on the lower leg is modulated by postural demands that can impact feedback processing for balance control, we asked participants to stand within a virtual room, facing a wall that would move towards them with low or high temporal uncertainty. This visual perturbation was introduced to challenge balance control, as has been shown in several previous studies^37–39^. When the perturbation is of high temporal uncertainty, and thus its onset cannot be easily predicted, we expect merely reactive postural adjustments. In contrast, when the perturbation is of low temporal uncertainty, and thus its onset is well-predictable, we expect anticipatory behaviour to the perturbations, and any reactive adjustments should be smaller compared to those elicited in the condition with a high temporal uncertainty. Importantly, to probe tactile sensitivity, we presented a brief tactile stimulus of varying intensity to the participant’s lower leg at various moments prior to and immediately after the onset of the visual perturbation, and participants had to verbally report whether they felt it or not. By presenting the probing stimuli to the skin over the calf area, instead of other areas, such as the foot sole, we not only assess how tactile input from that area is modulated by postural demands, but we also avoid confounds caused by pronounced skin deformations on the foot sole during postural sway that could themselves influence tactile perception^40^. In addition, this approach is similar to examining tactile sensitivity during upper limb movements, such as grasping actions, when the tactile probe is delivered around the area of the involved effector but not directly to the skin area engaged in the task e.g., fingertip during grasping^1,13,41^. Here, we probed the skin over the calf muscle that is in close proximity to the direct contact area between body and floor (foot sole), bearing in mind that the calf area conveys important tactile signals that can improve body posture^35,36^. It is also important to note that the probing tactile stimuli are neither informative nor relevant for maintaining upright stance in our study.

To confirm that our manipulation had the expected effects on body posture, we examined whether postural sway was reduced in anticipation of perturbations with low temporal uncertainty^25^and whether body sway increased after the onset of either type of perturbation. To examine these, we first assessed the idiosyncratic body sway in a baseline, quiet stance condition, where no perturbation was presented, and we subtracted each individual’s baseline sway from that during the perturbation trials. With respect to tactile modulation, we expected altered tactile sensitivity during perturbation compared to quiet stance trials. The main purpose of our study is to examine whether postural demands change the reliance on tactile feedback processing from the lower limb, and thus whether tactile sensitivity is temporally modulated during postural control. Based on findings from goal-directed arm movements^16,18^, we expected a temporal modulation reflected in increased tactile sensitivity at moments when postural demands are high, such as around the time of a postural reaction, where one needs to maintain posture in the presence of perturbation. Finally, we explored if any temporal modulation in tactile sensitivity differs between the two types of perturbations (high vs. low temporal uncertainty). In a second experiment, we explored whether the requirement to keep upright imposes any further changes in tactile sensitivity on the lower limb. To this end, we tested whether tactile sensitivity changes between standing and sitting.

## Results

Participants stood in a virtual room with the instruction to fixate a circular target on the front wall. In two separate blocks of trials, they were confronted with a visual perturbation that entailed the complete room moving in a way that the front wall approached them. Each of these blocks included perturbations that were initiated with a low uncertainty (i.e. after the presentation of a countdown) or high uncertainty manner (i.e. no countdown). A vibrotactile probe was applied to the skin of the participants’ calf at three possible moments during each trial: at one of 2 time windows before the onset of the perturbation, or at one time window right after the onset of the perturbation (*early*: −2.25 to −1.50 s; *late*: −1.50 to −0.75 s; *after*: 0.00 to 0.75 s; all relative to perturbation onset). Participants had to verbally respond if they felt the vibration, and we used the responses to determine detection thresholds based on an adaptive method (QUEST) applied separately per condition and participant. The following results focus on the participants’ behavior assessed by the center of pressure (COP) and their head kinematics, and on tactile perception.

### Experiment 1

#### Kinematics

**Fig. 1:**
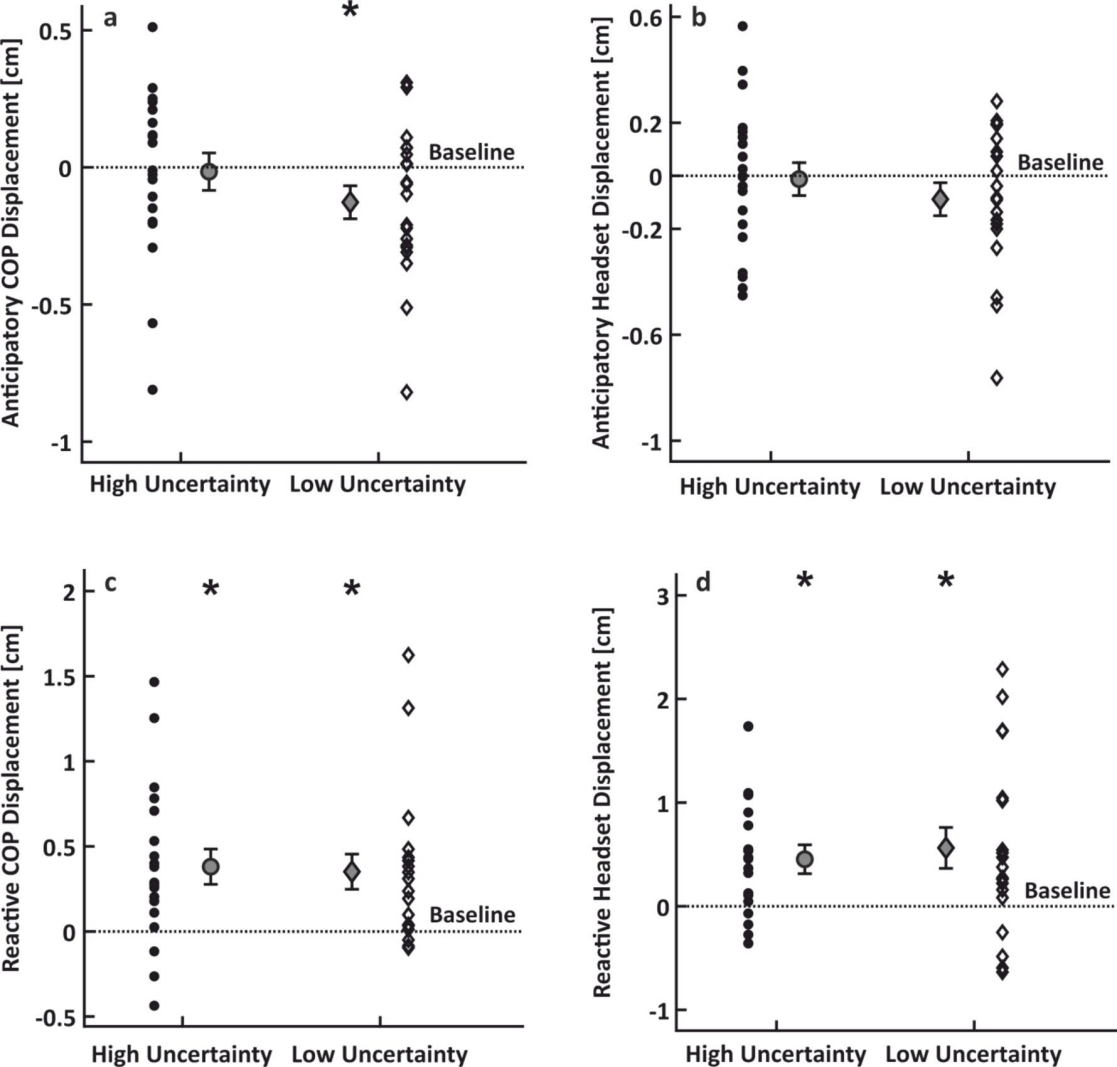
Results of the kinematic analysis. COP and head kinematic results for the anticipation and reaction periods. Panels a and b show anticipatory COP and head kinematic behavior, respectively. Panels c and d show reactive COP and head kinematic behavior, respectively. All four panels show single-subject data for the high uncertainty (circles) and low uncertainty (diamonds) conditions, as well as their corresponding means and standard errors. Dashed lines represent the baseline value as this was obtained during a quiet stance block with no perturbations. The * indicates *p* < 0.05 to tests against baseline values.

As expected, anticipatory postural behavior was generally evident prior to the low uncertainty perturbation compared to the respective baseline. Specifically, the maximal displacement of the COP was smaller during the period before the expected visual perturbation than during the respective quiet stance baseline (*t*_19_ = 2.114, *p* = 0.024, *η*^*2*^ = 0.053; Fig. 1a). This is in line with our hypothesis based on previous work^25^. However, the head’s maximal displacement before the expected visual perturbation was not systematically different from that during baseline (*t*_18_ = 1.420, *p =* 0.086, *η*^*2*^ *=* 0.026; Fig. 1b). Meanwhile, and as expected, there was no evidence of anticipatory behavior prior to the high uncertainty perturbation, as reflected both in the COP and the head maximal displacements (both *t* < = 0.202, both *p > =* 0.842, both *η*^*2*^ < = 0.001). These findings confirm that participants considered the underlying dynamics of the perturbations and predictively tuned their posture whenever possible.

Unsurprisingly, postural sway was larger when reacting to the low uncertainty perturbation relative to quiet stance, as reflected both in the COP (*t*_*18*_ = 3.402, *p* = 0.002, *η*^*2*^ = 0.132; Fig. 1c) and the head kinematics (*t*_*19*_ = 2.849, *p* = 0.005, *η*^*2*^ = 0.097; Fig. 1d). Likewise, sway amplitudes were larger during the reaction to the high uncertainty perturbation relative to quiet stance, evident both in the COP (*t*_*19*_ = 3.664, *p* < 0.001, *η*^*2*^ = 0.144) and the head kinematics (*t*_*19*_ = 3.261, *p* = 0.002, *η*^*2*^ = 0.117). These results indicate kinematic compensations of the visual perturbation in both perturbation conditions.

## Tactile sensitivity

**Fig. 2:**
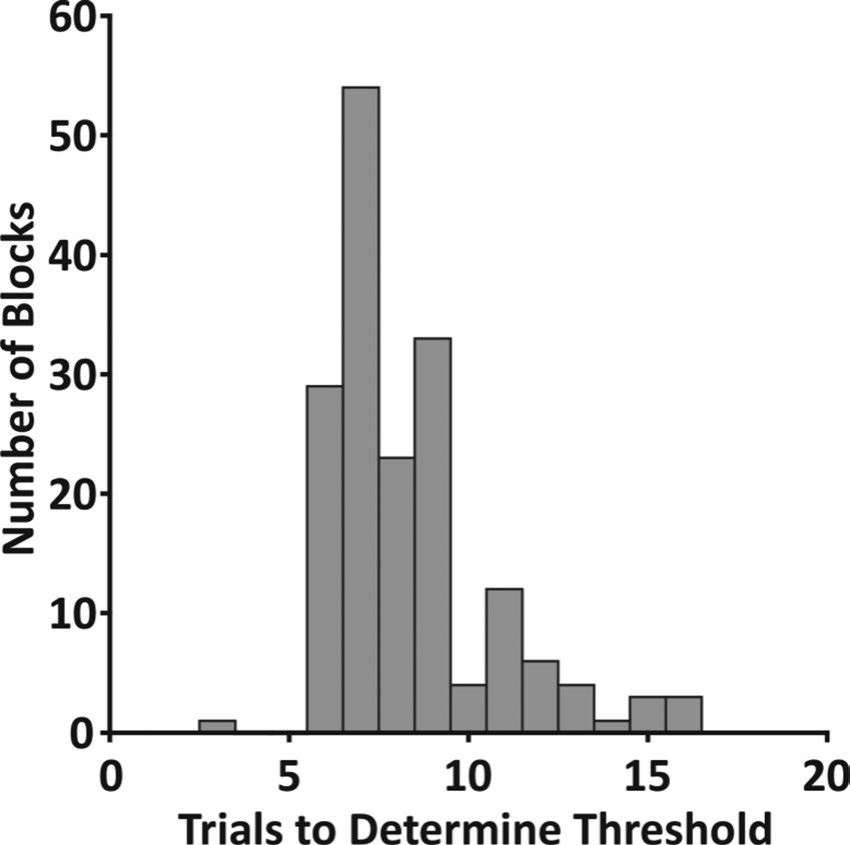
Trials until threshold estimation. Histogram of the required number of trials to determine all valid detection thresholds over all participants. The histogram shows cumulative how many trials it took to estimate the 173 detection thresholds obtained in all conditions of the experiment.

We assessed tactile sensitivity by calculating tactile detection thresholds through an adaptive procedure (QUEST). Figure 2 shows the distribution of the number of trials required for the QUEST algorithm to converge to the estimated detection threshold, for each of the 173 estimations. We presented 30 trials per block, and the average number of trials to estimate the detection threshold was only 8.3 (SD: 2.3). Thus, the number of trials per block was enough to obtain a detection threshold.

**Fig. 3:**
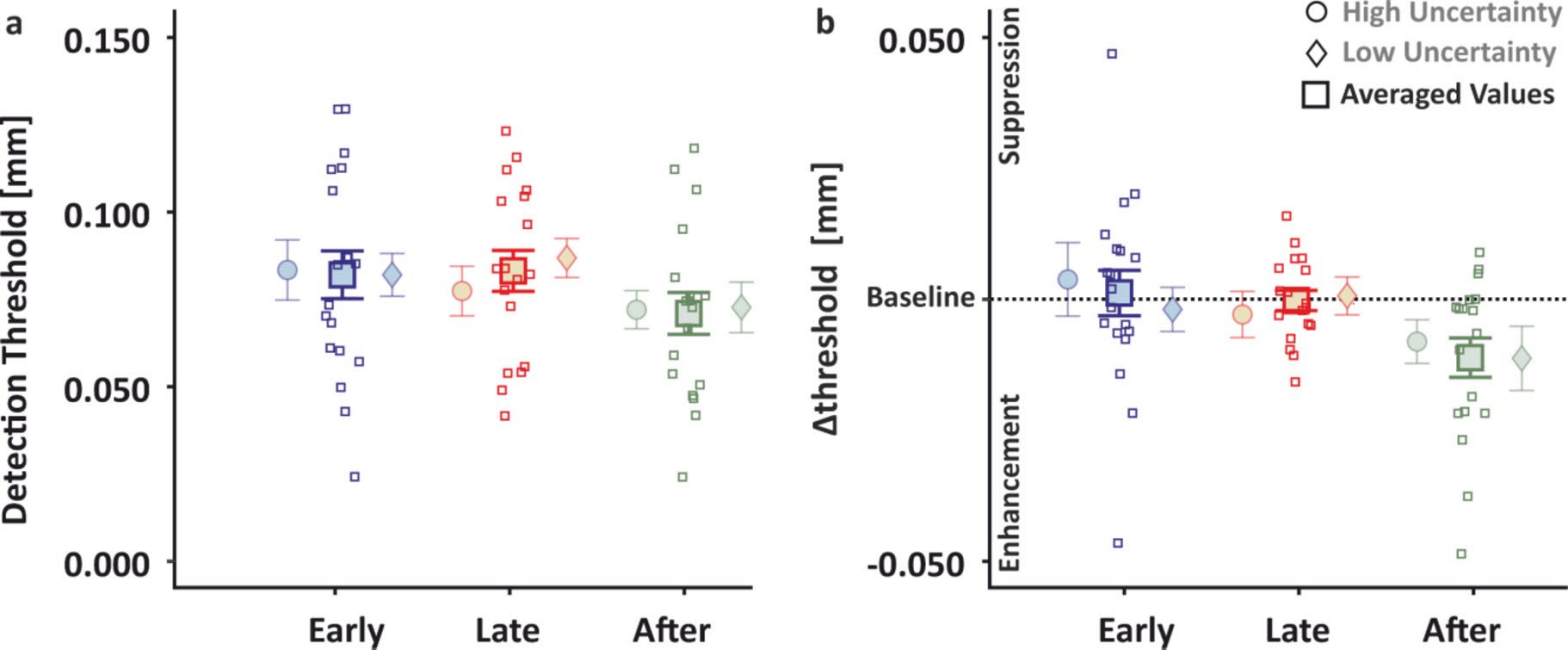
Psychophysical results Experiment 1. Detection thresholds during the perturbation blocks. Comparison of raw (**a**) and normalized to standing (**b**) detection thresholds for the 3 time intervals of the high (circles) and low (diamonds) uncertainty conditions are shown shaded with their average presented in darker hues in-between (square). Single subject data (squares) for the average are depicted. Error bars with the means and standard errors are shown.

Raw detection thresholds for each of the 3 time intervals during the perturbation blocks are depicted in Fig. 3a. Although some detection thresholds during the perturbation blocks appear to differ from those obtained during the respective standing baseline, on average the detection thresholds during perturbation blocks were not systematically different from those obtained during baseline (all *t* < = 1.933, all *p* > = 0.069, *η*^*2*^ < = 0.047).

Tactile perception during perturbation blocks relative to the standing (quiet stance) baseline (which we hereafter refer to as *Δthreshold*) was temporally modulated as reflected in a main effect of time (*F*_2,28_ = 4.364, *p* = 0.022 *η*^*2*^ = 0.054; Fig. 3b). Post hoc t-tests revealed significantly lower detection thresholds in the “after” compared to the “early” interval (*t*_18_ = 2.942, *p* = 0.019, *η*^*2*^ = 0.064), but there were no statistically significant differences for the thresholds between the “late” and “after” intervals (*t*_18_ = 1.699, *p* = 0.201, *η*^*2*^ = 0.022), nor between the “early” and “late” intervals (*t*_18_ = 1.243, *p* = 0.224, *η*^*2*^ = 0.012). These indicate that tactile sensitivity improved during the period immediately after the onset of the visual perturbation, at least relative to the “early” pre-perturbation period. There was no main effect of temporal uncertainty (*F*_1,14_ = 0.364, *p* = 0.556, *η*^*2*^ = 0.012) nor an interaction between uncertainty and time (*F*_2,28_ = 0.190, *p* = 0.828, *η*^*2*^ = 0.004).

## Experiment 2

**Fig. 4:**
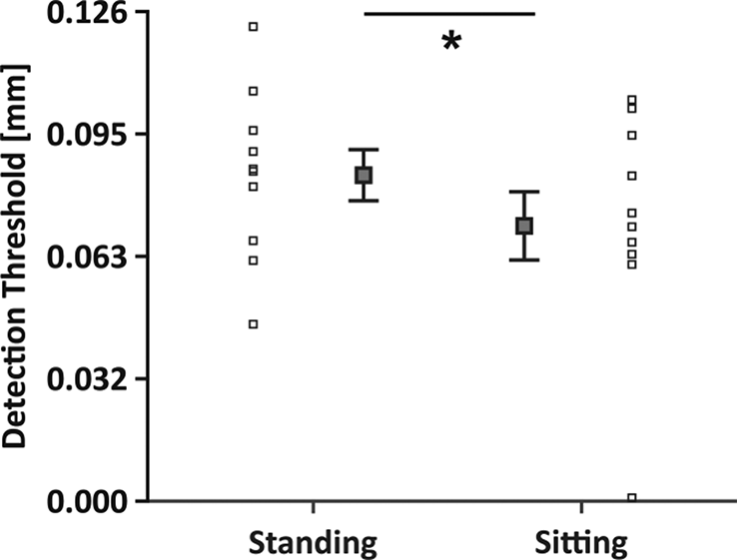
Tactile perception between standing and sitting. Comparison of raw detection thresholds between standing and sitting. This graph shows the detection thresholds for (quiet) standing (left) and sitting (right) baselines. Single subject data is depicted with squares, whereas averages and standard errors are shown with squares and the respective error bars. * indicates *p* < 0.05.

We conducted Experiment 2 to test if standing already causes an elevation of the tactile detection thresholds, which might marginalize any possible additional effects of feedback processing demands on tactile detection thresholds during the perturbation trials as was previously shown for foot sole stimulation^42^. To this end, we first contrasted tactile sensitivity during quiet stance against a new sitting baseline. Indeed, standing itself led to reduced tactile sensitivity (*t*_9_ = 2.285, *p* = 0.048, *η*^*2*^ = 0.115; Fig. 4), in line with previous work^42^.

**Fig. 5:**
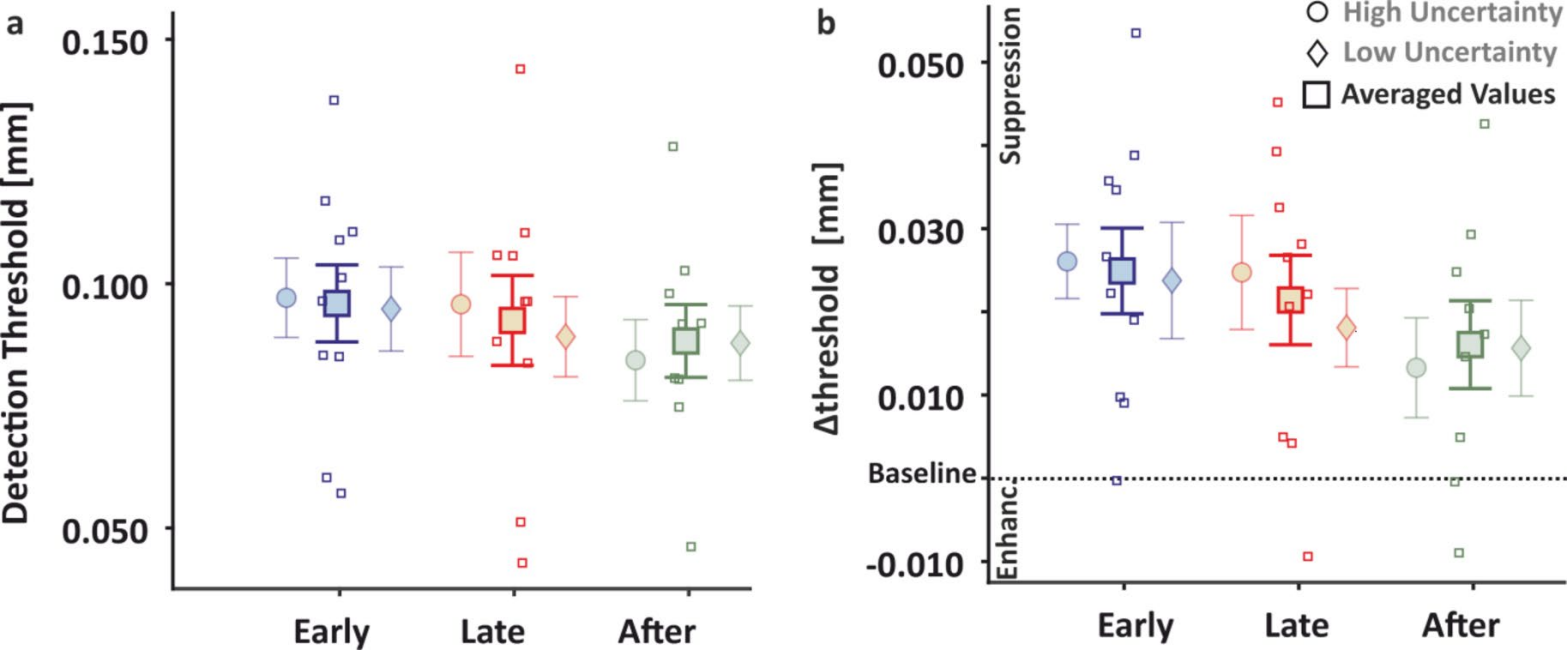
Psychophysical results Experiment 2. Detection thresholds during the perturbation blocks. Comparison of raw (**a**) and normalized to sitting (**b**) detection thresholds for the three time intervals of the high (circles) and low (diamonds) uncertainty conditions are shown shaded with their average presented in darker hues in-between (square). Single subject data (squares) for the average are depicted. Error bars with the means and standard errors are shown.

Raw detection thresholds during the 3 time intervals of the perturbation blocks in Experiment 2 are shown in Fig. 5a. On average these thresholds were higher than the detection thresholds obtained in the sitting baseline (all *t* > = 2.213, all *p* < = 0.027, all η^2^ > = 0.109; Fig. 5b). These show a clear decline in tactile sensitivity during standing in the perturbation trials compared to sitting.

As in Experiment 1, the 3 × 2 ANOVA on Δthresholds revealed a main effect of time (*F*_*2,16*_ = 16.159, *p* < 0.001, *η*^*2*^ = 0.247; Fig. 5), showing that tactile sensitivity was temporally modulated while anticipating and reacting to perturbations. Specifically, tactile detection thresholds were reduced in the “after” compared to the “early” (*t*_*8*_ = 5.455, *p* < 0.001, *η*^*2*^ = 0.108) and “late” (*t*_*8*_ = 4.113, *p* = 0.002, *η*^*2*^ = 0.064) intervals, and did not differ between the “early” and the “late” intervals (*t*_*8*_ = 1.342, *p* = 0.171, *η*^*2*^ = 0.07). These are in line with the findings of Experiment 1 and demonstrate improved sensitivity at times around the time of perturbation, thus at moments when sensory guidance of posture gains importance. There was again no main effect of temporal uncertainty (*F*_*1,8*_ = 1.089, *p* = 0.327, *η*^*2*^ = 0.037), nor an interaction (*F*_*2,16*_ = 0.642, *p* = 0.539, *η*^*2*^ = 0.024).

## Discussion

We examined whether tactile sensitivity on the lower limb is modulated by feedback processing demands. To this end, we used an adapted version of the classical moving room paradigm^38,39,43,44^ and examined the temporal modulation of tactile sensitivity on the lower leg during standing, while preparing for and reacting to visual perturbations that occurred at moments of low or high temporal uncertainty. We measured tactile sensitivity on the lower leg at different times during this task. We demonstrate that feedback processing demands modulate tactile sensitivity, enhancing sensitivity at the moments around the onset of the reactive postural response. This demonstrates that tactile sensitivity on the standing leg is temporally tuned to the dynamics of the perturbation and the need to sample sensory feedback from the probed body part to allow for balance retention.

The increased tactile sensitivity on the lower leg after the onset of the perturbation indicates an upweighting of somatosensory feedback signals from the standing limb around that moment. This appears inconsistent with previous findings that found *reduced* tactile sensitivity right before and during single finger movements^41,45^, reaching and grasping actions^16^, or foot extensions^19^. This apparent discrepancy can be explained by differences in the task relevancy of the sensory information from the probed limb. In our study, somatosensory information from the lower leg is important to control posture, particularly around the onset of the reactive response. This aligns with findings showing increased somatosensory-evoked potentials to tactile sensations from the foot sole shortly before initiating a step^20^. Such sensory modulation could reflect a reweighting of sensory signals based on their contribution to the underlying task, a phenomenon observed in various sensory modalities when maintaining an upright stance^23,24,46^. In our experiment, postural control relies on multiple sensory inputs, including visual, vestibular, and somatosensory signals. Since vestibular and somatosensory cues remain aligned, any conflict introduced by visual perturbation renders vision unreliable. This could prompt an increased reliance on other sensory channels, such as tactile input, to maintain balance. In a similar paradigm, a previous study reported that visual signals are up-weighted and perturbed proprioceptive signals from the leg are down-weighted when anticipating the avoidance of an approaching ball^25^. Though this might appear as inconsistent with our results, it is noteworthy that the down-weighting of proprioceptive inputs referred to Achilles tendon vibration signals that caused illusory postural sway, and that down-weighting such signals may be beneficial when maintaining balance. The modulation of tactile input from our participants’ lower leg might also be related to the perceived threat related to the upcoming collision and the retention of body balance. For instance, improved tactile sensitivity around the time of the postural response might be caused by increased feedback processing associated with fear of the approaching wall, a possibility that would align with research on threat-induced modulation of tendon reflexes^21,47^. However, it is unlikely that this factor played a major role in our study, as participants were not only told that the wall would not hit them, but they likely learned after a few trials that the wall would not make contact with them. Taken together, we suggest that the sensorimotor system can flexibly up- and down-weight somatosensory signals depending on how reliable information they can provide for postural state estimation.

We also examined the effect of temporal uncertainty related to the moment when the visual perturbation was triggered. There is evidence that sensorimotor uncertainty can influence the strength of tactile sensitivity^14,48,49^. Here we explored if low temporal uncertainty about the onset of the perturbation would lead to stronger reliance on predictive control when postural demands increase. Although we found anticipatory postural behavior before perturbations of low temporal uncertainty, especially reflected in a reduction of the COP sway, we have no evidence that tactile sensitivity was influenced as well. We cannot exclude the possibility that the uncertainty about the onset of the perturbation was too small to impact tactile perception since the general trial structure of the two perturbation conditions was comparable. Alternatively, the variance in the perception data could be too high to detect meaningful changes, but different between the two uncertainty conditions. One factor contributing to the variance is that participants were allowed to either stand with or without socks on the force plate. As the type of footwear was kept constant across condition, this should not have influenced our results, but it might have impeded the detection of the uncertainty effect. Temporal uncertainty of the perturbation may have also influenced the between-subject-variance through anticipatory effects, such as a reduction in variance under low uncertainty. However, the observed variances appeared comparable across conditions and experiments.

Interestingly, in Experiment 1, tactile sensitivity during standing was not different between trials involving perturbations and those without (i.e. quiet stance). This indicates that standing upright already leads to increased tactile detection thresholds. Indeed, when compared to sitting in Experiment 2, tactile sensitivity during standing was significantly reduced, in line with previous findings that assessed tactile sensitivity on the foot sole^42^. Tactile suppression during upright stance may stem from one or more sources. One possibility is that muscle activation or changes in leg position altered skin stretch by affecting subcutaneous volume, which may have changed the skin’s mechanical properties around the probed area and reduced tactile sensitivity^50^. For instance, skin deformation is known to influence tactile perception with higher deformation reducing tactile perception thresholds^40^. It is also possible that backward masking from the activation of the lower leg muscles, as well as the pressure on bones, and the tension of ligaments, tendons, and muscle spindles, increased sensory noise and thus made the brief vibrotactile probes less conspicuous. Indeed, standing itself is accompanied by an oscillatory movement^51^and postural adjustments^52^. When the stance is less stable e.g., the base of support is reduced, the activity of the lower leg muscles increases^53^ and this increase in peripheral activity may mask the probing tactile stimuli^9,2^. However, so far there is no clear evidence that increased force production and hence muscular activity per se can modulate tactile suppression^10^.

The sitting baseline occurred always at the end of the experimental procedure. This might render the reduced tactile sensitivity during standing as a by-product of improved tactile detection performance at the later (sitting) baseline simply due to practice. This could lead to lower detection thresholds towards the end of the experiment compared to the preceding standing tasks. This is unlikely in our experiments for two reasons. First, the standing baseline tasks that were presented at the end of Experiment 1 did not yield lower detection thresholds than the earlier presented perturbation tasks. Thus, the lower detection thresholds during sitting, which was the last block in Experiment 2, are unlikely to arise simply because of a longer exposure to the tactile task. Second, tactile detection thresholds, at least on the hand, do not differ between sessions separated by up to 30 min^15^. All in all, we have no evidence that the reported effects may be caused by a possible improvement in tactile detection over the course of the experiment.

Our kinematic results demonstrate small but systematic anticipatory behavior prior to the perturbations when temporal uncertainty was low. Unsurprisingly, there were no anticipatory adjustments prior to the perturbation of high temporal uncertainty. This is in line with previous work showing anticipatory behavior prior to predictable perturbations^25,54^. The anticipatory effects were mostly evident in the COP, while the head kinematics followed a similar but more variable pattern. This might be due to the sensorimotor system prioritizing the stabilization of the head over the trunk, as head stabilization is a common biological strategy. Stabilizing the head could reduce transformations of the sensory signals triggered during head movements or could provide a stable reference frame for the task at hand^55^. We also confirmed that participants react to new visual information by compensatory COP and head adjustments in the direction of perturbation evident after the onset of the room’s motion, and independently of its temporal uncertainty in line with previous literature^38,44^. The reactive amplitudes are rather small and well within the base of support, but it is important to note that our participants had a narrow base of support (i.e., feet close together), which would make it disadvantageous to exert large COP excursions that could lead to postural instability and possibly loss of balance. Indeed, when the base of support induces greater postural instability, body reactions have much smaller amplitudes^31^.

Together, our results demonstrate dynamic temporal modulation of tactile sensitivity on the lower limb when maintaining whole-body balance around visual perturbations and broaden our understanding of tactile sensory integration into postural control. The temporal tuning of tactile sensitivity when anticipating and reacting to a visual perturbation provides evidence for the idea that tactile sensitivity is dynamically modulated to the feedback demands. This upweighting of sensory feedback signals can lead to improved tactile sensitivity on a moving body part when somatosensory feedback signals from that part gain relevance for the sensory guidance of the movement.

Understanding the processing of tactile information in uncertain environments can be leveraged to assess and monitor an individual’s ability to control posture. Recognizing the dynamic nature of tactile perception, rather than categorizing it as merely sharp or poor, provides a deeper insight into postural instability. Our results expand the set of studies showing that tactile sensitivity can be modulated during an action [e.g.^14–16,18,20^, especially when feedback signals from the probed limb are important for the task, suggesting that tactile modulation around human movement is a dynamic process.

### Limitations of this study

Even though there were anticipatory behaviors before the low uncertainty perturbation and no adjustments before the high uncertainty perturbations, we did not detect differences in tactile sensitivity between these two conditions. We assume that the uncertainty was large enough to cause small postural adjustments but too low to elicit measurable changes in tactile sensitivity. Another limitation relates to the origin of the tactile modulation. While we demonstrate that tactile sensitivity is dynamically modulated in both experiments, our experimental paradigm cannot pinpoint the exact underlying mechanisms behind this phenomenon. We describe possible explanations for this phenomenon in the Discussion.

Due to the low spatial specificity in the modulation of tactile perception^56^ we were able to probe tactile perception on the lower leg. It is possible that the observed effect on increased sensitivity just before reactive adjustments would be even stronger and an anticipatory increase in tactile sensitivity could be observable when probed on the foot sole as done in other studies^20^. However, our device did not allow for such testing, opening an interesting avenue for future research.

## Method details

### Experiment 1

#### Participants and apparatus

We recruited 20 participants (24.4 ± 4.8 years old; range: 19–39, 11 ♀ 9 ♂) for a one-session virtual reality experiment. One additional participant was recruited but did not finish data collection due to circulatory problems. Participants were free from any known neurological or musculoskeletal issues at the moment of the experiment and had normal or corrected-to-normal vision. All participants signed an informed consent. The study was approved by the local ethics committee of the Justus Liebig University Giessen. All methods were carried out in accordance with the “World Medical Association Declaration of Helsinki” (2013, except for § 35, pre-registration)^57^. At the end of the experiment, participants received for their efforts either 8€/hour or course credits.

A custom-made vibrotactile stimulation device (Engineer Acoustics Inc., Florida, US), which will be referred to as a “tactor”, was attached to the skin area over the *musculus gastrocnemius lateralis* of the participant’s right calf. It comprises a small housing (22 × 45 × 5 mm) and a round actuator (6 mm in diameter) that can generate vibrotactile stimuli of fine-grained duration, frequency, and amplitude (for vibrations of 250 Hz, this is between 0.00316 and 0.632 mm). This tactor was fixed at 3 cm medial of 2/3 of an imaginary line connecting the heel with the *caput fibulae* using kinesio tape. Thus, the tactile device was fixed on that specific area to serve as proxy of tactile signals when maintaining upright stance, considering that previous works has demonstrated that tactile input from the lower leg can provide important signals for postural control^36^. Compared to the foot sole or the Achilles tendon, this area is low in skin deformity which is a known cofounder of tactile perception^40^. With additional tape, care was taken that loose wires or clothing could not be mistaken as vibrations and that they would not hinder the performance of the task. While surface vibrations have been shown to modulate the function of the Golgi tendon organs and muscle spindles, their effect is likely minimal due to the high stimulation frequencies and low indentation amplitudes involved^58,59^. Furthermore, these alterations in afferent signalling should not interfere with the detection of tactile input from the surface.

**Fig. 6:**
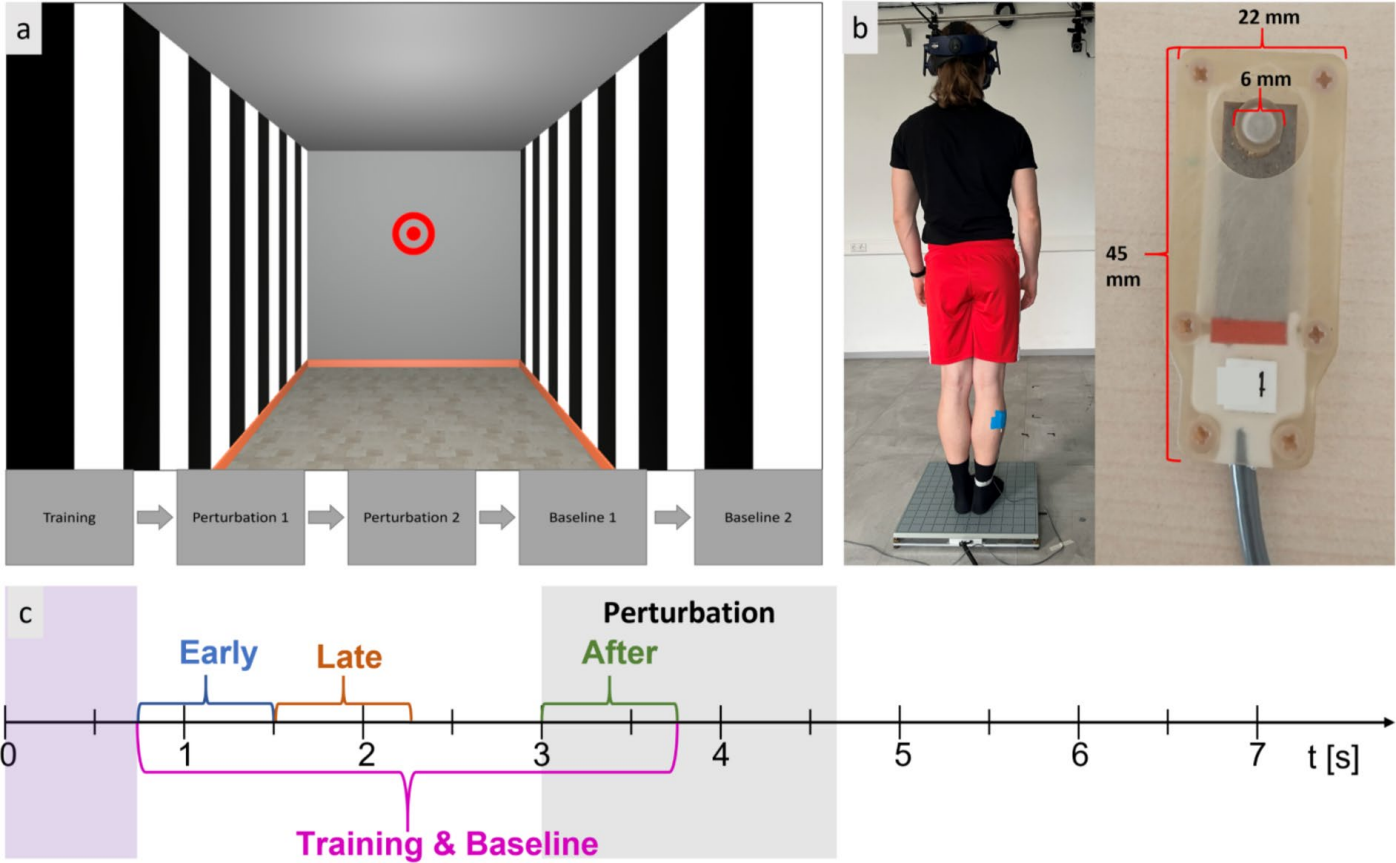
Experimental setup. (**a**) Depiction of the virtual environment as seen from the participants with the block order below. The red circular fixation point appears at the front wall of the virtual room. (**b**) Setup in the lab with a photo of the tactor and its size in mm. (**c**) Schematic timeline of a single trial of the perturbation condition. The probe tactile stimulus in the two perturbation blocks could be presented at one of three possible intervals: “early” (blue), “late” (brown), and “after” (green), whereas in the baseline and training conditions, it could be presented at any moment during a single interval (purple). The onset of the perturbation is depicted here as starting at the 3rd second of the trial, but this could vary in the condition with the high uncertainty perturbation. The duration of the perturbation is indicated by the grey area.

Participants stood barefoot or with their socks on having their feet closed together on a force plate (AMTI, Massachusetts, US) that sampled the center of pressure (COP) at 300 Hz. They also wore a head-mounted display (HTC Vive Pro Eye, Taiwan) that presented a virtual environment and sampled translatory movements at a rate of 90 Hz. Participants were then immersed in a 3 × 3 × 6 m (width, height, depth) room with black and white striped side walls and a grey front wall. At 1.85 m above the floor, a circular fixation point with a 60 cm radius was presented (Fig. 6). The virtual environment was a custom-made Unity version 2021.3.4f1 (Unity Technologies, San Francisco, US) build. Kinematic and ground-reaction force data were synchronized using Vicon Nexus 2.11 and streamed into Matlab via the Vicon DataStream SDK 1.11 (Vicon Motion Systems, Oxford, UK). The Unity environment and headset data were synchronized by transmitting a trigger via LabStreamingLayer 1.14, achieving a latency of less than 0.1 ms on a local machine.

### Procedure

The experiment consisted of five separate blocks of trials, each lasting 7 s. The first block was always composed of 30 training trials. Here, a countdown (3, 2, 1) appeared above the fixation point on the front wall, with each digit presented for 0.75 s. At the end of this countdown, a visual perturbation occurred, which consisted of the complete room moving in such a way that the front wall approached the participant with a velocity of 3 m/s for 5 m. Thus, the movement of the room toward the participant lasted for 1.67 s. The room size and motion were based on a previous study designed to elicit postural sway in participants^44^. After each trial, the room instantly recovered back to its original configuration. After this training block, two perturbation conditions were presented in a random order across participants. In each of these two blocks, the room could be perturbed at a moment of low or high temporal uncertainty. In the low uncertainty condition, trials started with a fixed delay of 0.75 s after the experimenter pressed a button, followed by a 2.25 s period of countdown, and the visual perturbation occurred immediately after that countdown. In other words, the perturbation was expected based on the presented countdown and it always occurred 3 s after the onset of the trial. In the high uncertainty condition, a trial would start without a countdown, and the perturbation would occur within a random moment between 2.25 and 3 s from the beginning of the trial. The absence of the countdown and the 0.75 s random delay increased the uncertainty about the exact moment of the perturbation onset. Everything else, including the velocity of the room’s movement, were identical between the two levels of uncertainty. These resulted in a difference in the uncertainty of when the perturbation would occur between these two conditions, while also allowing us to capture the same time window during which the countdown appeared. Thus, we could examine possible anticipatory sensorimotor responses prior the perturbation in both perturbation conditions. Upon completion of these two perturbation conditions, two blocks followed where we assessed baseline sensory and motor performance: one block with and another block without a visual countdown, but importantly both blocks without any visual perturbation. In other words, these two baseline conditions were identical to the two perturbation conditions but now the virtual room never moved. The order of the two baseline conditions was identical to the order of the perturbation conditions, and this order was randomized across participants. Participants were explicitly informed prior to the onset of each block about the condition that they would be exposed to. In all blocks, participants were asked to fixate the fixation point at the center of the front wall at all times with their head in a natural configuration.

To probe tactile sensitivity, we delivered a brief (50 ms, 250 Hz) vibrotactile stimulus of varying intensities at the skin of the participants’ right lower leg, over the calf muscle. The stimulus was presented at various moments during each trial, and participants had to verbally report whether they felt a vibration or not. This tactile stimulus is not relevant for the postural task, but is used as a proxy to assess tactile sensitivity; a procedure commonly applied in previous work^1,16,18^. In the two perturbation conditions, the probing tactile stimulus was delivered at a random moment during one of three possible intervals relative to perturbation onset: two at the pre-perturbation period (“early”: −2.25 to −1.50 s, and “late”: −1.50 to −0.75 s) and one at the post-perturbation period (“after”: 0.00 to 0.75 s; Fig. 6). We presented 30 trials at each of these three intervals in a randomized order, for a total of 90 trials per perturbation block. These three intervals were chosen based on the following considerations: tactile sensitivity during the “early” interval should be mainly unaffected by predictive mechanisms. The “late” interval should test whether predictive processes associated with the onset of visual perturbation will tune tactile sensitivity differently in the low and high uncertainty conditions. Finally, the “after” timepoint should cover any modulation that is primarily caused by the visual perturbation itself and the motor reactions.

In each of the training and two baseline conditions there was a single time interval during which the tactile probe could be presented: for the training and baseline condition with the countdown the stimulus was presented between 0.75 and 3.75 s, whereas for the baseline condition without the countdown it was presented between 0 and 3.75 s, always relative to the onset of the trial.

The intensity of vibration in each trial was determined using a QUEST algorithm (Psychtoolbox Version 3.0.18). As a Bayesian approach, the QUEST algorithm uses prior knowledge to estimate the parameters of a psychometric function. As the model function we used a Weibull distribution because it can represent a wide range of response patterns. We chose a β of 3.5 for the distribution which is the suggested value for 2 AFC tasks in the QUEST algorithm^60^. The prior mean and standard deviation for the training block were estimated from a pilot study where participants just stood with their feet closed (*N*= 16, exposed to 50 stimuli of varying intensities, presented at the skin over the calf muscle of the right leg). We used the mean of the estimated probability density function as probe intensity for the next trial. For all the other conditions we used the estimated threshold of the training block as prior mean^60,61^.

In each trial, participants were instructed to verbally report whether or not they felt a vibration. They were instructed to immediately respond once they felt a vibration to avoid memory effects, but they were also explicitly asked to respond after the trial ended if no answer was meanwhile given. These verbal responses were then registered by the experimenter via button presses to a computer, which induced a short delay (0.5–1 s) between trials. To prevent fatigue, participants could have a break after every trial on request. Breaks of ~ 1 min were enforced every 30 trials and longer breaks of ~ 5 min were introduced after 90 trials. During these longer breaks, participants were required to sit and were allowed to remove the headset. This resulted in a duration of around 20 min for each perturbation block, with a total experimental time of 90 min. Trial control, data acquisition, and data analysis were handled by custom-made Matlab R2021a software (The Mathworks, Massachusetts, US).

### Data analysis

The COP and headset translational data were analyzed only in the anterior-posterior (negative to positive values, respectively) direction as the visual perturbation happened in this direction. In a first step, we excluded trials from all kinematic and psychophysical analyses with data that were continuously missing for at least 100 ms (1.2%; only COP data were ever missing). Afterward, we normalized every sample of each trial to the average position of the first 0.5 s of that trial, which we consider as a period of quiet stance because there was nothing happening to the room. The resulting normalized data was corrected for outlier data points by replacing values with a displacement larger than 15 cm with no value (this only affected COP data), and then smoothed utilizing a symmetric moving average with a window size of 100 ms.

**Fig. 7:**
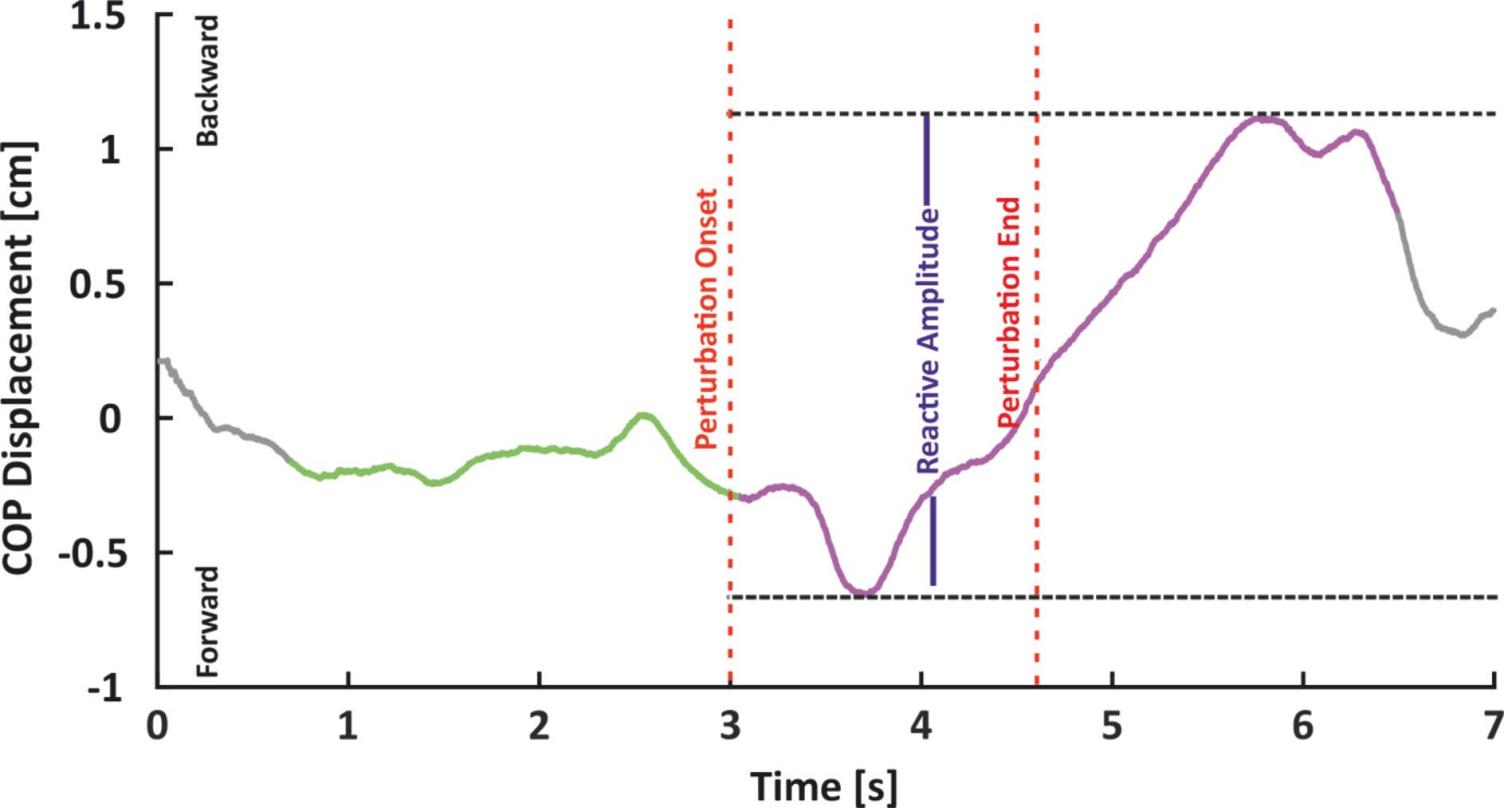
Exemplary kinematic data. Temporal profile of the center of pressure (COP) time course in the antero-posterior direction of a low uncertainty trial. The time window of perturbation (red dashed lines), the anticipatory period (green path), the reactive period (purple path), and the reactive amplitude (blue solid line) are depicted.

Anticipatory behavior were quantified as the maximum absolute COP and head amplitude during the last 2.25 s prior to perturbation onset, that is the time window from the first possible vibrotactile probing until reactive movements could appear. To quantify reactive kinematic behavior we first determined the maximum positive (posterior) COP and head displacement during the first 3.5 s following the onset of the perturbation. This corresponds to about twice the perturbation duration. Determining the maximum positive COP and head position values to quantify motor responses was important because any reaction to the perturbation would result in the body leaning backward and thus to a positive COP and head position value. We then quantified reactive kinematic adjustments of the COP and head as the difference between their maximum value and their respective minimum that occurred between the perturbation onset and that maximum (see Fig. 7). We calculated both components for every trial of each subject and averaged across all trials for each uncertainty level. Both of the previous measures were normalized with respect to the values calculated from their respective baseline periods (quiet stance) to account for individual differences in standing. Thus, values greater than zero indicate larger displacements during the perturbation blocks relative to the respective baseline.

We determined tactile detection thresholds for each condition and participant. This was quantified as the first trial after which the probe stimulus intensity recommended by the QUEST did no longer change value. It is important to note that our device accepts only integer values as inputs for the tactile stimuli, but the values recommended by the QUEST could also include decimals. In these cases, we always rounded the suggested value to the nearest integer. Thus, although the QUEST suggested slightly different intensities in the trials after the detection threshold was determined, we presented the same stimulus intensities due to rounding. We expected some fluctuation in the responses to those trials due to natural randomness in detecting stimuli around one’s own detection threshold. For this reason, we considered detection thresholds as invalid if participants gave the same (detected or not-detected) response in the remaining trials after the detection threshold was determined. The thresholds of those blocks (4.2%) as well as the corresponding kinematic data were excluded from further analyses. To quantify tactile modulation during perturbation relative to quiet stance trials while also accounting for individual differences in tactile sensitivity, we subtracted each participant’s baseline detection threshold from their threshold obtained at each time interval of the respective perturbation block (Δthreshold), a procedure in line with previous work^10,13^. To closer align the kinematic with the perceptual results we included in the kinematic analyses of each condition and participant only those trials that were used to estimate the associated detection thresholds.

### Statistical analysis

We first examined whether anticipatory behavior was evident prior to perturbation compared to baseline by testing if the maximum displacement in COP and head kinematics during the anticipatory period deviated from the respective values during quiet stance. Because we subtracted the maximum displacement in baseline from the respective value in the perturbation blocks, we quantified possible effects by using one-sided one-sample t-tests against zero for the low uncertainty (based on the directed hypothesis) and two-sided t-tests for the high uncertainty condition (based on the undirected hypothesis). Similarly, we tested whether the reactive component in each perturbation condition deviated from zero (i.e. baseline) using one-sided one-sample t-tests.

To first explore possible modulation of tactile sensitivity during the perturbation blocks relative to baseline, we submitted the obtained Δthreshold values to six separate two-sided one-sample t-tests against zero (i.e. baseline). Our main interest was whether tactile perception would be temporally tuned while anticipating and reacting to a perturbation. We also explored whether there would be any effects of the temporal uncertainty of the perturbation. To test for possible effects of the time interval of tactile probing (“early”, “late”, “after”) and the type of perturbation (low vs. high uncertainty) on tactile sensitivity, we used a 3 × 2 repeated measure ANOVA. Significant main effects were explored with post-hoc t-tests, with p-values corrected for multiple comparisons wherever necessary using the Holm procedure, and with all t-values reported as absolute values. The type one error threshold was set to 0.05 and effect sizes are reported as *η*^2^ following the calculations and recommendations of Correll and colleagues^62^. Statistical analyses were conducted in JASP version 0.17.1 (University of Amsterdam, Netherlands). Datapoints in any of the 6 experimental or 2 baseline conditions that were outside of the 3.5 interquartile range were excluded from the statistical analysis of that condition as outliers (< 1% for kinematic and 0% for psychophysical data). If any baseline value was excluded, the three values in the associated perturbation condition were also excluded since normalization was impossible.

### Experiment 2

In Experiment 1 we examined the temporal modulation of tactile sensitivity when coping with visual perturbations of high or low temporal uncertainty during upright stance. To this end, we compared tactile perception when standing and being confronted with a perturbation to when standing without having to cope with any perturbation. However, standing itself may already influence tactile sensitivity on the leg, for instance by masking feedback signals, or by changes in skin and muscle-related properties on the probed body part. To account for such possibilities, we conducted Experiment 2, where we examined whether tactile detection thresholds at the lower leg differed between quiet stance and sitting. We further examined whether tactile modulation followed similar patterns to those observed in Experiment 1.

### Participants and experimental design

We recruited 12 new participants, with two of them not finishing data collection due to circulatory problems or incompliance with task instructions. Thus, our final sample was 10 participants (25.3 ± 4.0 years old; range: 21–32; 9 ♀ 1 ♂) who joined a one-session experiment. The procedure was almost identical to Experiment 1, but participants now performed six blocks of trials: a training block, two perturbation blocks, two standing baselines, and after these, an additional sitting baseline. During this sitting baseline, participants wore the head-mounted display and sat relaxed on a chair with their knees flexed at ~ 90° and both feet touching the floor. In this sitting baseline, participants performed 30 trials that were constructed identically to those in the other two standing baseline blocks. In all six blocks of Experiment 2, participants saw the same room as in Experiment 1. The experimental procedure and analyses were identical to those reported for Experiment 1, except for the details mentioned below.

### Data and statistical analysis

As we were interested in the modulation of tactile sensitivity by standing, we focused our analysis on tactile detection thresholds. In contrast to Experiment 1, detection thresholds obtained during the two perturbation blocks of Experiment 2 were now normalized with respect to the new sitting baseline, resulting once again in six Δthreshold values per participant. The analysis of the participants’ responses after the detection threshold resulted in excluding 1.5% of the total detection thresholds and their corresponding kinematic data. Furthermore, and since we did not observe any differences in the two standing baselines in Experiment 1 (*t*_19_ = 0.621, *p* = 0.542, *η*^*2*^ = 0.005) or Experiment 2 (*t*_9_ = 0.263, *p* = 0.798, *η*^*2*^ = 0.002), we now averaged across the two standing baselines thresholds for each participant.

A two-sided paired t-test was performed to test for differences between the standing and the sitting baselines. To further examine whether tactile detection thresholds increase during the perturbation blocks relative to the sitting baseline, we contrasted the six Δthreshold values against zero (i.e. baseline) with six separate one-sided one-sample t-tests. Additionally, we submitted the Δthreshold values to a 3 × 2 repeated measure ANOVA to investigate for main effects of the time of probing (“early”, “late”, “after”) and of type of perturbation (low vs. high uncertainty) on tactile modulation, as in Experiment 1.

## Key resources table

**Table:** 

Resource	Source	Identifier
*Software and Algorithms*		
Matlab 2021a	MathWorks	https://de.mathworks.com/products/matlab.html
Vicon datastream SDK	Vicon Motion Systems	https://www.vicon.com/software/datastream-sdk/
QUEST	Psychtoolbox 3.0.18	http://psychtoolbox.org/
JASP 0.17.1	JASP	https://jasp-stats.org/
LabStreamingLayer	LabStreamingLayer	https://github.com/sccn/labstreaminglayer
Unity 2021.3.4f1	UNITY	https://unity.com/
*Hardware*		
Tactor	Engineering Acoustic Inc.	https://eaiinfo.com/
Forceplate	Advanced Mechanical Technology Inc.	https://www.amti.biz/
VIVE Pro Eye	HTC Corp	https://www.vive.com/de/

## Data Availability

Behavioral and psychophysical data are publicly available after acceptance at https://osf.io/jdzwa/. For further information or data requests please correspond to Fabian Dominik Wachsmann (Fabian.Wachsmann@psychol.uni-giessen.de).

## **Declarations**

### Resource availability

*Lead contact*.

Further information and requests for resources should be directed to and will be fulfilled by the lead contact, Fabian Dominik Wachsmann (Fabian.Wachsmann@psychol.uni-giessen.de).
